# Perspectives and preferences of domestic violence survivors regarding digital platform and AI chatbot for help-seeking: A qualitative study

**DOI:** 10.1371/journal.pone.0342453

**Published:** 2026-02-23

**Authors:** Vivian Hui, Lidan Tian, Xinyu Feng, Qiqi Chen, Arkers Kwan Ching Wong, Tina L. Bloom

**Affiliations:** 1 School of Nursing, The Hong Kong Polytechnic University, Hong Kong; 2 Health and Community Systems, School of Nursing, University of Pittsburgh, Pennsylvania, United States of America; 3 Department of Applied Social Science, The Hong Kong Polytechnic University, Hong Kong; 4 School of Nursing, Notre Dame of Maryland University, Baltimore, Maryland, United States of America; Macao Polytechnic University, MACAO

## Abstract

**Background:**

Domestic violence (DV) is a pervasive public health issue with profound physical, psychological, and social consequences. Help-seeking, particularly access to advocacy interventions, plays a pivotal role in promoting resilience and mental health well-being among DV survivors. Digital platforms and AI chatbots are emerging as promising tools for information and support. However, limited research has explored the views and needs of DV survivors regarding these technologies, impeding the development of culturally sensitive interventions for help-seeking.

**Objective:**

This study aimed to investigate the perspectives and preferences of digital platform and AI chatbot among people with DV experience in Hong Kong, which could offer valuable insights to inform the design of tailored technological solutions for help-seeking.

**Methods:**

Semi-structured qualitative interviews were conducted with 36 individuals who had DV experience over the past decade. Interviewees were recruited from DV-specific non-governmental organizations (NGOs) in the community using convenience and snowball sampling. Interviews took place either in-person or via videoconference between April 2023 and August 2024. Data were analyzed using reflexive thematic analysis, combining deductive and inductive approaches. Three trained coders identified themes and sub-themes through an iterative and collaborative process.

**Results:**

We identified three key themes regarding the recommended features of digital platforms: functions, content, and format. Regarding content, participants valued evidence-based information, step-by-step guidance, and survivor narratives. For format, participants valued procedural visualization, engaging multimedia, calming visual design, and clear text layout. Participants emphasized the potential of AI chatbots, highlighting benefits such as enhanced accessibility, efficient information filtering, and providing nonjudgmental feedback. However, concerns were raised about chatbots’ limitations, particularly in empathy, personalization, and information accuracy.

**Conclusions:**

DV survivors preferred digital platforms that prioritize safety, accessibility, and emotional support. Our study highlights the role of AI as a complementary tool to human support and calls for participatory approaches to facilitate designing effective interventions to reach their full potential.

## Introduction

Domestic violence (DV), or family violence, is a pattern of abusive behavior used by one member to gain or maintain power and control over another member within the same household. This includes intimate partner violence (IPV), child abuse, and elder abuse, encompassing physical, sexual, economic, and psychological harm. DV has been recognized as a complicated public health issue for decades, primarily affecting millions of families around the world [1]. Exposure to DV often results in significant physical and psychological consequences, including traumatic brain injuries, chronic pain, unwanted pregnancies, post-traumatic stress disorder, depression, anxiety, suicidal ideation [2–6]. These health impacts not only decrease quality of life for DV survivors but also contribute to a non-harmonious society with high risks of mental health issues, thereby placing a significant burden on healthcare systems [7,8].

Help-seeking, particularly access to violence-specific advocacy interventions, is one of the key protective factors that foster resilience and mental health well-being among family violence survivors [9]. For example, IPV-focused advocacy interventions empower IPV survivors by providing support, resources, and guidance—such as legal aid, housing, safety planning, and health services—delivered by trained mentors, professionals, or volunteers in various forms, durations, and intensities [10]. Nevertheless, survivors’ barriers to such services include feelings of shame, guilt, stigma and fear of judgment [11,12]. Additionally, some can be re-traumatized or encounter misjudgment by service providers [13,14]. In Hong Kong, DV is often viewed as a “family affair,” and cultural values emphasizing family privacy may create additional barriers to disclosure and help-seeking [15].

Recent advancement in digital intervention has significantly transformed the potential for accessible, private, and scalable solutions tailored to the unique needs of DV survivors. A recent integrative review highlighted the surging use of health information technology and scoping review pinpointed artificial intelligence (AI) techniques in uncovering DV information and emotional needs across different datasets, such as online health communities, clinical narratives, police narratives etc. [16,17]. This evidence suggested that technology-based interventions can deliver tailored support to DV survivors,including training modules, legal information, safety planning strategies and emotional supports [18]. Another recent systematic review [19] of 17 randomized controlled trials found modest but significant effects of digital and technology‐based IPV interventions on depression (at 3 months), anxiety (at 3 months), and physical victimization (at 6 months) among female survivors. This review included several widely-adopted digital platforms, notably the MyPlan app, developed by Glass et al [20], which provides screening, danger assessment, education, and prioritization activities for IPV survivors. This app has been adapted for diverse global settings, with variants including myPlan Kenya [21], I-DECIDE (Australia) [22], and iSafe (New Zealand) [23], alongside iCAN Plan 4 Safety in Canada [24]. Additionally, mobile-based interventions have shown effectiveness in reducing suicidal ideation and hopelessness in patients with major depressive disorders [25], supporting their potential to positively influence mental well-being for vulnerable populations.

The technology has evolved from stand-alone websites, mobile applications, videoconferencing, to simulation and chatbots [16]. Digital platforms, with their multi-functional capabilities, offer a comprehensive approach by combining resources, emotional support, safety planning, and real-time assistance into a single ecosystem, making them more accessible and holistic for violence survivors. However, while these digital tools have demonstrated significant potential, they typically lack a key component of advocacy interventions for violence survivors, which is empathic support [10].

AI chatbots may offer a promising alternative, as they can provide individualized and anonymous support that addresses DV survivors’ need for confidentiality [19]. There is growing evidence supporting the applications of generative AI in mental health support. Research shows that ChatGPT can identify PTSD symptoms by analyzing trauma narratives [26]. Generative AI has also been applied to trauma-related treatment, enabling personalized interventions and improved access to care [27]. A recent systematic review found that generative AI-powered chatbots demonstrated remarkable effects in alleviating mental distress among adolescents and young adults [28]. The rise of large language models (LLMs) facilitated the growth of research in this spectrum. LLMs, is one of the sub-groups of AI, with their advanced natural language processing capabilities, provide a robust foundation for developing AI chatbots that can engage in empathetic, context-aware, and culturally sensitive conversations with users. Previous literature demonstrates the potential of LLM technologies in providing emotional support, guidance and improving mental health outcomes among patients with post-traumatic stress disorder, postpartum depression, bipolar depression etc. [29,30]. These LLM technologies have the potential to bridge gaps in traditional help-seeking mechanisms for DV survivors, particularly those with limited access to in-person advocacy. Survivors in underserved areas may face geographical barriers to professional services, while others may avoid seeking help due to shame, stigma, or fear of judgment [31]. LLMs can address these gaps by offering anonymous and nonjudgmental interactions, around-the-clock availability, and consistent responses that reduce the risk of re-traumatization often associated with in-person disclosure [32,33].

Despite the growing trend of using LLM techniques to improve mental health in western countries, there is a paucity of research that explored the possibilities of LLM/AI interventions in DV populations, and a significant gap regarding culturally-congruent digital violence interventions [19]. Recent work by Guan et al. [31] demonstrated that LLMs can accurately classify the information needs of DV survivors based on their online posts, suggesting the feasibility of applying such technologies to this population. However, no LLM-based intervention has yet been developed specifically to support DV survivors’ help-seeking. To inform the design of such interventions, it is crucial to first understand survivors’ perspectives and preferences regarding the functionality and features of help-seeking digital platforms and AI chatbots. Yet, to our knowledge, no prior study has investigated this topic. This gap is particularly important within the context of Chinese culture, which is deeply influenced by patriarchal structures and traditional Confucian values. These cultural factors often discourage open disclosure of traumatic DV experiences due to concerns about familial harmony, and social stigma. Effective uptake of advocacy interventions depends on how well they fit survivors’ circumstances and cultural context [10], and understanding survivors’ perspectives is of paramount importance for designing culturally sensitive and effective digital interventions. Therefore, the overarching goal of this study is to explore the perspectives and preferences of digital platform and AI chatbot for help-seeking among people with DV experience in the Chinese context. Specifically, the research questions were formulated as follows:

RQ1: What are the perspectives and preferred features for building digital platform for help-seeking among participants with DV experience?

RQ2: What are the perspectives and preferred features for AI chatbot for help-seeking among participants with DV experience?

## Methods

### Study design

This is a qualitative descriptive exploratory study to explore the perspectives and preferences of digital platform and AI chatbot for help-seeking among people with DV experience in Hong Kong.

### Setting and participants

Participants were recruited using a convenience sampling approach and snowball sampling through word-of-mouth. During April 2023 to August 2024, participants were mainly recruited from DV-specific non-governmental organizations (NGOs) in the community, including Harmony House, and Caritas Family Crisis Support Centre, as well as online social media through Facebook ads. Promotional flyers were sent to NGOs and the staff referred potential research participants for our study. Each interested participant was screened by a research assistant via phone call to ensure their eligibility and safety to join the research study.

The inclusion criteria are: (1) aged 18 years or above; (2) people who have experiences DV over the past ten years; (3) living in Hong Kong and (4) proficient in Chinese or English. The exclusion criteria include: (1) under 18 years old; (2) non-Hong Kong residents, (3) DV experiences happened more than ten years (4) a clinical diagnosis of cognitive disorder including Alzheimer’s disease, amnesia, and delirium or have any psychiatric disease that cannot provide accurate information, and (5) failure to give informed consent. The ten-year timeframe was chosen to encompass the rapid digitalization of DV support services, during which help-seeking resources transitioned from predominantly traditional face-to-face services to diverse digital modalities. Survivors over this period were exposed to those approaches, positioning them to provide insights into how digital platforms could enhance traditional services.

### Data collection

Originally, we targeted 40 participants for in-depth interviews. Nevertheless, we reached data saturation at 36 individual interviews, determined through iterative analysis when no new themes emerged, and the research team decided to terminate the recruitment to avoid duplicated information for analysis. Hence, we conducted (N = 36) individual interviews among participants who have experienced DV through in-person interviews or Zoom interview. The interviews leveraged a semi-structured format with open-ended questions, focusing on participants’ perspectives on DV-specific digital platform and AI chatbot for better help-seeking experience. This study is part of the main study to explore the help-seeking behavior and online help-seeking behavior among DV survivors. To ensure clarity and consistency in understanding, the Principal Investigator (PI) provided explicit definitions of key terms, including “digital platforms” and “AI chatbots,” during the interview process. Digital platforms referred to web-based or mobile applications designed to provide DV information, safety planning, danger assessment, and referrals services for individuals experiencing DV. AI chatbots referred to conversational agents that engage in text-based dialogue to provide emotional support, information, and guidance. The PI showed an example of digital platform (e.g., 1800repsect.org.au) and AI chatbot (e.g., GPT 3.5/ Llama 3) in front of participants. Participants were given five minutes to familiarize themselves with these examples before the interview questions. This step was taken to ensure that all participants shared a common understanding of these concepts, thereby enhancing the reliability and validity of the data collected. The interview questions were designed based on the help-seeking theoretical framework [34], which delineates three recursive stages in the help-seeking process: problem recognition and definition, decision to seek help, and selection of support sources. This framework informed our interview guide by structuring questions around participants’ experiences across these stages, exploring how individual, interpersonal, and sociocultural factors influenced their help-seeking behaviors. Specifically, questions addressed participants’ perceptions of traditional versus digital support modalities, barriers and facilitators to accessing each type of resource, and preferences for future digital interventions. This framework has also been applied in prior research to investigate online help-seeking behaviors within the same population [35]. The interview processes were guided by a faculty member who is a registered nurse with more than five years domestic violence research experience and assisted by two undergraduate nursing students. The principal investigator of the research study delivered 2 hours training for two nursing students to equip their interview skills for this vulnerable populations, such as prompting skills, active listening skills, asking open-ended questions without taking side, as well as trauma-informed approaches. Thereafter, two students were given opportunities to shadow the interview session hosted by the principal investigator multiple times before being the interviewer in reality. Each interview lasted about 45–60 minutes. All the interviews were subsequently recorded with a recording pen after obtaining the written consent and verbal permission from participants. As for the interview conducted virtually in Zoom software, the research team obtained their verbal consent after explaining the research study objectives, participants’ rights and mailed the written consent forms for participants’ written signature subsequently. Participants were reminded not to disclose their personal information such as names, areas of residence. Additionally, they were advised to turn off their cameras if they did not feel comfortable for the sake of protecting their privacy. The interview guide is provided in S1 Appendix.

As interviews were conducted in Cantonese, key findings and selected quotes were translated into English by a bilingual team member and verified by a second bilingual member, with discrepancies resolved through discussion.

### Data analysis

This study employed reflexive thematic analysis for investigation in order to identify and interpret themes inductively, providing a nuanced understanding to accommodate a wide range of DV survivors’ perspectives [36–38]. This approach emphasizes continuously questioning the data and emerging interpretations. It values the insights from researchers’ active and engagement with the data, rather than striving for a single objective outcome. This flexibility allows for a deeper, contextually grounded understanding of participants’ experiences, perceptions and behaviors [37,38]. All interviews were audio-taped, de-identified and transcribed by an external third-party vendor. To ensure transcription accuracy, three students independently reviewed the transcripts. The analysis followed a dual-phase approach: an initial deductive analysis using a predetermined set of codes derived from help-seeking theoretical framework, followed by an inductive analysis to identify emergent codes generated from the data.

The inductive coding process initially involved line-by-line open coding and discussions between the three students for the first 10 transcripts. Afterward, the three students reviewed and revised the codes and themes through focused coding of the first 10 transcripts and 10 additional transcripts. After each round of approximately 10 transcripts, the coding team held weekly meetings to review emerging patterns and assess whether new perspectives appeared in the data. Codes were organized into broader categories and structured hierarchically into sub-themes and main themes through bi-weekly meetings. The faculty member (VH) participated in team discussions to review coding decisions and theme development. After the 30th interview, the team observed that subsequent interviews were yielding perspectives consistent with the developed thematic framework. When subsequent new interviews offered no new insights, we concluded that the data had been saturated with 36 interviews. NVivo software was utilized to streamline the coding process and expand the manual coding to all 36 transcripts.

### Ethical considerations

The study has been approved by the Institutional Review Board at the Hong Kong Polytechnic University (HSEARS20230111007–01). All participants were fully informed about the study objectives. Informed consent was obtained through written signatures for in-person interviews or mailed consent forms for online interviews. As recalling DV experience could be traumatic to participants, they were guaranteed the right to withdraw from the study at any time point without any consequences, and multiple schedule breaks could be allowed for emotional healing if necessary.

## Results

### Overview of participants

In this qualitative study, we conducted in-depth interviews with 36 participants who had experienced domestic violence (DV). Most participants were female (75%, n = 27). The majority of participants were aged between 18–40 years (72.2%, n = 26), and over two-thirds had completed tertiary education (69.4%, n = 25). Parental violence was the most common form of DV experienced (55.6%, n = 20), followed by intimate partner violence (38.9%, n = 14). Almost all participants reported experiencing emotional/psychological abuse (94.4%, n = 34), often alongside physical abuse (80.6%, n = 29). When asked about their self-perceived identity, 47.2% (n = 17) identified as survivors, 30.6% (n = 11) as victims, and 22.2% (n = 8) as both victims and survivors. Demographic information is presented in Table 1.

**Table 1:** Demographic characteristics of participants (N = 36).

Characteristics	N (%)
**Gender**	
Female	27 (75)
Male	8 (22.2)
Prefer not to disclose	1 (2.7)
**Age group (years)**	
18-30	14 (38.9)
31-40	12 (33.3)
41-50	8 (22.2)
51-60	2 (5.6)
**Educational Level**	
Secondary School	11 (30.6)
Associate degree/Diploma	5 (13.9)
Bachelor’s Degree	13 (36.1)
Master’s Degree or above	7 (19.4)
**Monthly Income (HKD)**	
≤ 10,000	15 (41.7)
10,001-20,000	13 (36.1)
20,001-30,000	3 (8.3)
30,001-50,000	5 (13.9)
**Marital Status**	
Single	21 (58.3)
Married	5 (13.9)
Divorced	3 (8.3)
Separated	7 (19.4)
**Having Children**	
No	23 (63.9)
Yes	13 (36.1)
**Housing Type**	
Public Housing	17 (47.2)
Private Housing	6 (16.7)
Village Houses	4 (11.1)
Others (dormitory/company property)	9 (25.0)
**Perpetrator Type**	
Parental Violence	20 (55.6)
Intimate Partner Violence	14 (38.9)
Sibling Violence	2 (5.5)
**Types of Abuse Experienced** ^a^	
Emotional/Psychological Abuse	34 (94.4)
Physical Abuse	29 (80.6)
Economic Abuse	14 (38.9)
Sexual Abuse	2 (5.6)
**Self-identified Status**	
Survivor	17 (47.2)
Victim	11 (30.6)
Both Victim and Survivor	8 (22.2)

^a^ Percentages may sum to more than 100% as participants could select multiple types of abuse

### Perspectives and preferences of digital platforms

**Fig 1:**
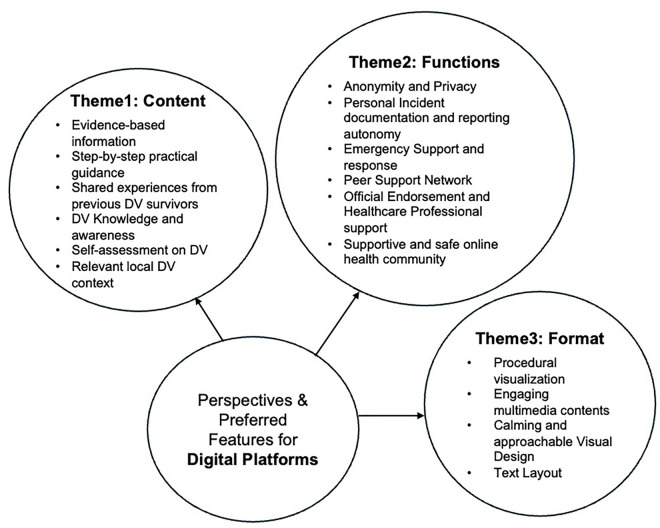
Thematic map of perspectives and preferred features for digital platforms.

This study explored participants’ recommendations for digital platform features supporting DV survivors. The thematic analysis identified three main areas: content, functions, and format. In content recommendations, participants emphasized the need for practical, evidence-based information organized in accessible formats, particularly step-by-step guidance and authentic survivor stories. For platform functions, participants prioritized safety features including anonymity protections, documentation tools, and emergency response systems, while also valuing integration with professionals and community support features. Format preferences revealed the importance of visual design elements that create psychological safety, alongside clear information organization and locally relevant content. These features were considered essential elements in developing effective digital support platforms that meet the unique needs of people who have experienced DV in Hong Kong. The relationship between main themes and sub-themes related to digital platform features is presented in Fig 1. The complete thematic analysis with all participant quotes is available in S2 Appendix.

### Theme 1: contents features of digital platforms

#### Evidence-based information.

Participants expressed strong interest in having data-driven and research-based information on digital platforms. They perceived digital platforms should not only provide direct support services but also serve as informational resources offering up-to-date numerical information. Multiple participants articulated a clear preference for empirical data and scholarly content, suggesting that they view such information as essential components of a credible and authoritative DV support platform.

“I enjoy reading things with numbers and data, like research reports” [p04]

“I’m interested in statistics about child-rearing, or spousal abuse, or sexual violence” [p26]

#### Step-by-step practical guidance.

Participants identified practical guidance as an essential content element. They emphasized the need for clear, sequential instructions that could guide them through complex processes such as legal procedures, safety planning, and family coping strategies. While acknowledging that individual situations might require different approaches, participants valued structured guidance that could provide a starting framework for action. As some participants stated:

“I want more practical steps, like what I need to do to file for divorce, step by step, or how to teach children to accept this family situation, or how to accept their past” [p10]

“It’s really important to have a step-by-step process, though such steps might not apply to every person’s case, but it still serves as a useful reference.” [p32]

#### Shared experiences from previous DV survivors.

Participants strongly valued authentic narratives from individuals with lived experience of DV. They expressed particular interest in recovery journeys and long-term outcomes, suggesting that survivor stories serve multiple functions: providing hope, practical insights, and motivation for those in similar situations. Many participants noted that peer narratives could reduce isolation and validate their experiences in ways that professional information alone cannot. As some participants stated:

“ Something that could be highly interactive, where I can see the experiences of other survivors or victims, especially seeing the results after they sought help. That might increase my motivation” [p05]

“ I’d like to know about people who experienced domestic violence as children, and what their mental state might be like when they reach their thirties or forties, or how they face building their own families” [p03]

#### DV knowledge and awareness.

Participants highlighted the need for clear definitions and explanations of DV. They specifically requested detailed categorizations and clear definitions of different types of abuse, suggesting that many individuals may struggle to identify or name their experiences. This emphasis on definitional clarity indicates that improved awareness and vocabulary around DV could be an important first step in the help-seeking process, allowing individuals to more accurately identify and articulate their experiences before seeking targeted assistance. As one participant suggested:

“The definition of domestic violence should be more detailed, categorized into physical violence, verbal violence, sexual violence, cold violence, and not just simply grouped under domestic violence” [p34]

#### Self-assessment on DV.

Participants suggested including structured assessment tools to help users evaluate their situations objectively. They particularly emphasized the value of screening tools for identifying DV experiences and understanding whether certain behaviors constitute abuse, which could help individuals overcome potential denial or normalization of abusive behaviors. Notably, one participant, reflecting on her childhood experience of not recognizing abuse, recommended that age-appropriate assessment tools should also be developed for children and adolescents to help them identify violence and understand when to seek help.

“If really designing a website, I think there should be some tests for them to do. I think these screening forms are very important, especially for children, to track if they have experienced domestic violence through their behavior, to let them know what they’re experiencing” [p27]

#### Relevant local DV context.

Participants emphasized the importance of culturally relevant and locally specific information within digital platforms for DV support. They expressed frustration with existing resources that often contained information from other regions that was not applicable to Hong Kong’s unique social, legal, and healthcare environment. As some participants explained:

“I noticed that a lot of information is from Taiwan, which isn’t very relevant to Hong Kong.” [p17]

“The information and titles should clearly indicate that they are specifically for Hong Kong.” [p18]

### Theme 2: functions features of digital platforms

#### Anonymity and privacy.

Participants emphasized the critical importance of maintaining user anonymity and privacy within DV support platforms. They articulated specific concerns about identifiable information requirements and expressed a strong preference for flexible privacy options that allow users to control their level of disclosure according to their comfort levels and safety situations. As participants stated:

“If the other person doesn’t mind, they can go public if they want to, or use a pseudonym if they prefer” [p02]

“What we should avoid is requiring full Chinese names, date of birth, or anything that can identify you” [p04]

#### Personal incident documentation and reporting autonomy.

The analysis revealed strong support for features enabling users to document their experiences privately and maintain control over reporting processes. Participants expressed interest in structured documentation tools that could help them record incidents while maintaining agency over next steps. As participants described:

“Maybe provide a simple Google Form where you can fill in what harm you experienced, how many times it happened, who the perpetrator was, when it happened, and then leave your contact information, so someone can contact you or your parents” [p10]

“There needs to be a place where help-seekers can write down what happened. Then they can choose whether they need immediate assistance” [p30]

#### Emergency support and response.

Participants identified immediate crisis response capabilities as a crucial platform function. They emphasized the importance of having 24/7 access to emergency assistance with clear response protocols and minimal barriers to urgent help, valuing both automated responses and rapid connections to human assistance. As some participants stated:

“I think they (1800RESPECT) did well with allowing immediate contact and stating it’s 24-hour service. I think this method is better because you can get an immediate response or clear guidance” [p16]

“Help-seeking needs to be quick. Whether it’s immediate online support or direct access to hotline numbers. It needs to be immediate because people are usually urgent when looking for these things” [p18]

#### Peer support network.

The analysis revealed strong interest in establishing structured peer support systems within the platform. Participants emphasized the value of connecting with others who have lived through similar experiences, suggesting both formal and informal peer support mechanisms. This function was seen as particularly important for creating sustainable, long-term support systems for survivors. Participants noted that peer connections could provide unique forms of validation and practical wisdom based on shared experiences, complementing professional support with experiential knowledge. As some participants described:

“They could hire or find volunteers who have experienced these things themselves to be comforters” [p23]

“Let victims or survivors share their stories. Then everyone can support each other, but this function needs to be promoted so that people who need it can find this channel” [p08]

#### Official endorsement and healthcare professional support.

Participants expressed a strong preference for platforms endorsed by official institutions and integrated with professional support services. They emphasized the importance of having access to registered social workers and healthcare professionals, suggesting that institutional credibility significantly impacts user trust and willingness to engage with digital support services. As one participant stated:

“Having government agencies endorse this, whether it’s the police department or even the Social Welfare Department” [p16]

#### Supportive and safe online health community.

The analysis revealed support for creating a moderated online space where survivors could safely share experiences and support one another. This emerged as a multi-faceted function combining peer support networks, professional moderation, and therapeutic sharing opportunities. Participants emphasized the importance of having dedicated spaces for survivors to document their recovery journeys and connect with others who understand their experiences, while also highlighting the need for active moderation to prevent harmful interactions that could re-traumatize users. As some participants stated:

“There should be a place for other survivors or victims to write about their experiences and how they recovered” [p10]

“It’s like having a discussion forum, but most importantly, it needs moderators. This prevents people from attacking each other or triggering each other’s trauma. When talking about these topics, people’s emotions tend to be more sensitive, and inner wounds can easily be triggered. Therefore, someone needs to manage the order” [p16]

### Theme 3: format features of digital platforms

#### Procedural visualization.

Participants highlighted the value of clear, step-by-step visual guides for navigating support processes. They expressed preference for interactive flowcharts and visual representations of procedures that break down complex processes into manageable steps. As one participant suggested:

“Should have diagrams showing the steps - like step 1 is making a call, step 2 is police report, and so on. You can click each step for more details” [p09]

#### Engaging multimedia contents.

Participants expressed strong preference for diverse media formats that combine text, visuals, and interactive elements, emphasizing the effectiveness of simulation videos and dramatized scenarios in making complex information about DV more approachable and comprehensive. As some participants shared:

“Like those simulation clips showing how domestic violence might happen” [p01]

“Find some actors to simulate the mindset of domestic violence victims and perpetrators, and then their housing and financial issues can be brief but hit the key points” [p11]

#### Calming and approachable visual design.

Participants valued visual elements that created a sense of safety and emotional support, specifically mentioning color choices and professional branding as important factors in establishing a supportive environment without triggering negative responses or trivializing DV experiences. As one participant expressed:

“Color matters too. Maybe more neutral, lighter colors that don’t give an aggressive feeling. Not too colorful as it’s not a happy thing, and no black as it feels too tragic” [p14]

#### Text layout.

Participants emphasized the importance of clear, well-organized text presentation, specifically mentioning the need for prominent display of emergency information and easily digestible content structure that avoids overwhelming blocks of text through strategies like chunking information and using bullet points. As some participants noted:

“‘Emergency Hotline’ should be in very large text, in an obvious location” [p14]

“Although it’s in list form like bullet points, if people see a long list like 1,2,3,4,5 but each point is still a large paragraph, it’s not concise enough” [p18]

### Perspectives and preferences for AI chatbot

**Fig 2:**
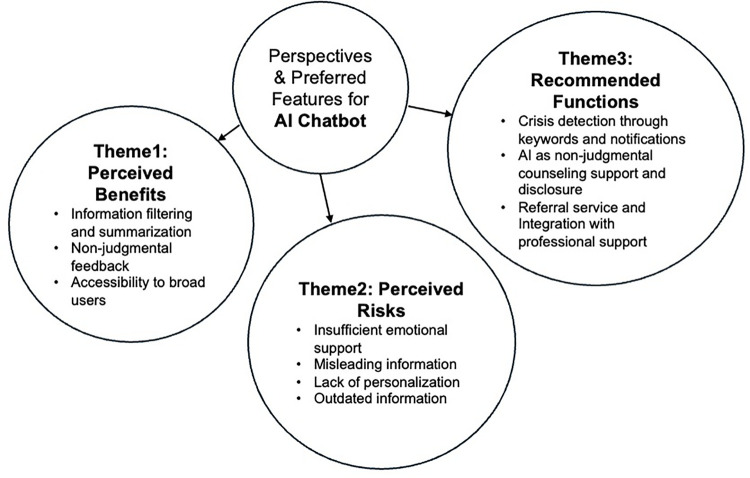
Thematic map of perspectives and preferred features for AI chatbot.

Participants generally believed that AI provides strong informational support, but simultaneously worried about the accuracy of information. The most concerning AI risk mentioned was the lack of emotional value that human support can provide. Views on data security were particularly complex. Some participants worried that AI would store their personal information due to the model’s training memory, while others believed that since AI is not human, it would at least not share their information with others. Fig 2 illustrates the thematic structure of participants’ perspectives on AI chatbot integration.

### Theme 1: perceived benefits

#### Information filtering and summarization.

Participants appreciated AI’s ability to process and organize large amounts of information efficiently. They highlighted its capability to filter complex information and provide concise summaries. Multiple participants mentioned the value of quick access to relevant information that AI could provide. As one participant stated:

“The chatbot can summarize and help you find the information you want, instead of googling and slowly typing keywords to search” [p34]

#### Nonjudgmental feedback.

Participants highlighted the value of AI’s neutral and unbiased approach to providing support. They specifically mentioned the importance of receiving feedback without feeling judged. Several participants noted that AI’s objectivity could be particularly helpful for sensitive discussions. As some participants expressed:

“ChatGPT’s best aspect is being nonjudgmental, as it can really speak to you in a very neutral way” [p28]

“It won’t judge me. Its evaluation is truly as a third party, it won’t have any bias, because when it speaks it seems a bit careful not to offend you, or it gives rational analysis from different angles” [p34]

#### Accessibility to broad users.

The analysis showed that participants appreciated AI’s potential to make services more accessible to diverse user groups. Participants noted the value of AI in reducing barriers to seeking help, particularly for those who might struggle with traditional support methods. As some participants mentioned:

“Some victims might not have the patience to read online information, or elderly people might not be literate. Through AI, demonstrations and explanations might be clearer and more interesting” [p11]

“ChatGPT is relatively simple in that the knowledge requirement isn’t that high. You might ask a question, and as long as you can type, that’s OK.” [p13]

### Theme2: perceived risks

#### Insufficient emotional support.

Participants expressed significant concerns about AI’s inability to provide authentic emotional support. They highlighted the lack of lived experience and emotional connection in AI interactions. Several participants emphasized that this limitation was particularly important in crisis situations. As participants stated:

“Because ultimately a robot just has numbers programmed into it, robots don’t have emotions and haven’t experienced anything firsthand” [p01]

“AI really can’t replace psychologists and social workers. I think it can help you find a website map more quickly, but responding to emotions would be difficult” [p27]

#### Misleading information.

Participants highlighted concerns about the accuracy and reliability of AI-provided information. They specifically mentioned worries about incorrect or irrelevant information, particularly regarding local services and resources. As some participants noted:

“I worry about the credibility of information provided by AI. Because its current search engine might sometimes combine information from different countries, it might give a helpline number that can’t be dialed in Hong Kong. I really worry about the accuracy of its information” [p05]

“I used ChatGPT to find information for me, but the results it gave me were fake” [p13]

#### Lack of personalization.

The analysis revealed concerns about AI’s ability to provide truly personalized support. Participants noted that AI responses might be too generic for complex personal situations. As one participant explained:

“If the case is very simple, then I believe ChatGPT can teach you what to do. Most times human-to-human issues might be the most complex and difficult to handle. Maybe when you ask ChatGPT, everyone might get almost the same information, so such a one-size-fits-all approach might not really work” [p32]

#### Outdated information.

Participants expressed concerns about the currency of AI-provided information. They specifically mentioned limitations related to database updates and real-time information. As some participants pointed out:

“It’s still using a lot of outdated data” [p15]

“Because I’ve used ChatGPT myself, but it’s not real-time - its database gets updated, so the information you can find is actually limited. That’s why I haven’t really used it for searching” [p34]

### Theme3: recommended functions for AI Chatbot

The analysis also identified several key functional recommendations for AI integration in DV support platforms. These recommendations focused on crisis detection, counseling support, and professional service integration. Participants emphasized the importance of maintaining human oversight while leveraging AI capabilities.

#### Crisis detection through keywords and notifications.

Participants suggested implementing AI-powered crisis detection systems. They recommended keyword monitoring and automated alerts for high-risk situations. Several participants emphasized the importance of early intervention for safety concerns. As some participants described:

“Suppose you might type certain words, then it’s a high red flag, meaning I know this case is very likely to be, for example, suicide” [p15]

“Perhaps when the system sees victims writing messages that show a lot of low self-esteem, or even suicidal tendencies. This system could use AI to identify these high-risk individuals” [p02]

#### AI as nonjudgmental counseling support and disclosure.

Participants highlighted the potential role of AI in providing initial counseling support and facilitating disclosure. They emphasized the value of AI’s nonjudgmental nature for users who might be hesitant to speak with human counselors initially. Several participants noted AI’s potential to create a safe space for initial disclosure. As some participants mentioned:

“I even think that if AI counseling develops better, it might know what to say at the right moment, which could make it even more sensitive to people’s needs” [p18]

“I’ve heard that some people around me, or friends of friends, would set keywords for these AI chatbots to treat them like therapists or something similar” [p37]

#### Referral service and integration with professional support.

Participants expressed support for integrating AI services with professional human support. Participants emphasized the importance of maintaining human backup and creating clear pathways to professional services. Several participants highlighted the need for seamless transitions between AI and human support. As some participants suggested:

“AI could function more like a personal emergency alarm system. There could be a platform with AI capabilities that can detect when there might actually be a fatal incident about to occur, then social workers would be alerted through the AI and could immediately make a phone call” [p01]

“It would be good if someone talks to AI first and then gets referred to relevant professionals. This helps them understand how to properly seek help and express what they want to do” [p23]

## Discussion

### Principal findings

This qualitative study explored perspectives and preferences regarding digital platforms and AI chatbots for help-seeking among individuals who have experienced DV in Hong Kong. Our findings reveal participants’ recommendations for digital platforms across three key dimensions of content, functions, and format. Participants emphasized the need for evidence-based information, step-by-step practical guidance, and authentic survivor narratives as essential content elements. For platform functions, participants prioritized anonymity protections, documentation tools, emergency response capabilities, and integration with professionals. Format preferences highlighted the importance of procedural visualization, engaging multimedia content, and calming visual design that creates psychological safety while maintaining professionalism.

Regarding AI integration, participants identified several potential benefits, including efficient information filtering, nonjudgmental feedback, and improved accessibility. However, they also expressed significant concerns about insufficient emotional support, potentially misleading or outdated information, and lack of personalization. Participants recommended AI functions focused on crisis detection through keyword monitoring, nonjudgmental counseling support for initial disclosure, and seamless integration with professional human services.

### Comparison with prior research

Our findings on AI chatbot integration in DV support reveal important parallels and distinctions when compared with research on AI applications for other trauma populations. One of the primary benefits of AI chatbots is their ability to provide nonjudgmental and anonymous support, which can be particularly appealing to trauma patients who may feel vulnerable discussing their experiences. Survivors in this study expressed that AI chatbots would be neutral and unbiased, valuing their nonjudgmental nature. Nevertheless, AI chatbots may exhibit biases embedded in their training data [39], underscoring the need for rigorous data curation when designing tools for sensitive populations like DV survivors. Siddals et al. [40] highlight the way this feature has allowed generative AI chatbots have emerged as a novel solution for mental health interventions, allowing users to engage in conversations that can facilitate emotional processing and coping strategies. Similarly, Saqib et al. [41] emphasizes that these chatbots can enhance therapeutic experience by offering personalized interactions that may encourage patients to disclose sensitive information more freely than they would in traditional settings. This aligns with findings from Pham et al., who discuss the potential of AI chatbots to deliver emotional coping mechanisms and support for individuals facing communication difficulties [42].

However, despite these advantages, extant research does suggest a palpable reluctance among some patients and healthcare professionals to fully embrace AI chatbots, and similarly, our participants had numerous concerns about the effectiveness, trustworthiness, or currency of the information chatbots might provide survivors, and wanted to see DV interventions including chatbots display official endorsement by trusted organizations. This is congruent with Nadarzynski et al’ report [43] that while there is a growing interest in AI-led chatbot services, many users remain cautious about their effectiveness and the quality of care they can provide. The emotional nuances of trauma recovery, which often require human empathy and understanding, may not be adequately addressed by AI technologies, leading to hesitancy in their adoption [44]. Moreover, the context in which these chatbots are deployed significantly influences user perceptions. Cheng and Jiang’s study [45] on AI-powered mental health chatbots in crisis situations suggests that users’ motivations and engagement levels can vary dramatically based on the circumstances surrounding their use. This indicates that while some patients may find solace in AI interactions during crises, others may prefer human interaction, especially when dealing with the aftermath of traumatic events.

Our findings strongly suggested that the integration of AI chatbot – like other digital interventions for violence survivors [19] are best seen as a complementary tool rather than a replacement for traditional therapeutic methods or for human interaction. Our participants were concerned about the lack of authentic emotional support, the ability of the chatbot to truly personalize information or detect crises, and expressed interest in connecting with other survivors. This perspective aligns with research by Miner et al. [46] suggesting that while AI can improve access to care, it cannot replicate the depth of understanding and emotional connection that human therapists provide. This sentiment is echoed by Rubin et al., who argue that empathy is a critical component of effective therapy, and while AI can simulate certain empathic responses, it lacks the genuine emotional intelligence that human therapists possess [47].

Additionally, ethical considerations and patient trust are paramount in the successful integration of AI into DV support. Awuah et al. stress the importance of transparent communication and ethical guidelines to build trust between patients and AI systems [48]. This is echoed by Benda et al., who highlight that understanding patient perspectives on AI is crucial for its acceptance and effective implementation in mental health care [49]. The need for robust ethical frameworks is further underscored by Hogg et al., who note that stakeholder perspectives are essential in shaping the future of AI in clinical settings [50].

The perspectives on AI chatbots among trauma patients are multifaceted. While these technologies offer promising avenues for emotional support, particularly in terms of accessibility and personalization, there are substantial concerns regarding their ability to replace human empathy and the nuanced understanding required in trauma care. As the field evolves, it will be crucial to address these concerns and ensure that AI chatbots are integrated thoughtfully into therapeutic practices, maintaining a balance between technological innovation and the essential human elements of care. For example, once initial screening and support have been provided by AI chatbots, human therapists can step in to offer more personalized and in-depth intervention such as building healthy therapeutic relationships, tailored treatment plans based on the insights gathered from AI interactions to better understand patients’ needs and progress, making in-person sessions more focused and productive.

### Implications for digital platform design

It is essential to acknowledge that many DV victims and survivors have experienced trauma, which profoundly influences their interactions with digital platforms. Our findings demonstrate that participants’ preferences for digital platform features including calming visual design, anonymity protections, and moderated community spaces directly reflect trauma-informed needs. Research indicates that trauma-informed design can create emotionally safer environments for users, particularly in digital health technologies [51]. Williams et al. highlight that trauma-informed practices can reduce the risk of re-traumatization and improve recovery outcomes for victim-survivors by aligning service delivery with their needs [52]. Furthermore, Owen and Crane discuss how the built environment, including digital spaces, can be designed to support mental health and mitigate the effects of trauma, particularly for those with high incidences of PTSD, such as domestic violence survivors [53]. Collectively, these findings underscore the value of trauma-informed design in creating safer digital support environments for survivors. Alongside these design considerations, it is important to recognize that digital platforms also carry potential risks that warrant careful consideration. Specifically, for survivors whose devices are monitored by abusers, inadequate platform privacy may expose their help-seeking activities and lead to further harm. Alternatively, exposure to distressing content or negative interactions may increase the risk of re-traumatization. To mitigate these risks, platform design could incorporate robust anonymity options, quick-exit buttons, content warnings, and professional moderation of peer communities.

Additionally, the range of features participants identified—such as documentation tools, safety planning resources, peer support communities, evidence-based information, and emergency response capabilities—serve distinct purposes and may not realistically be integrated into a single comprehensive platform. Documentation and safety planning tools require secure, private interfaces focused on individual crisis management, while peer support communities necessitate moderated social spaces that prioritize interpersonal connection. Similarly, emergency response features demand immediate accessibility and integration with professionals, whereas educational content benefits from browsable, repository-style designs. These varying functional requirements suggest that DV digital support may be better served through either modular platform designs that allow users to access different tools as needed, or through an ecosystem of specialized platforms that focus on specific support functions. Future platform development should consider how to balance comprehensiveness with usability, potentially through clear navigation systems that guide users to appropriate resources based on their immediate needs, or through interoperable platforms that can refer users across specialized services while maintaining continuity of care.

### Limitations and future directions

Several limitations should be noted. Our sample predominantly consisted of younger, educated female participants, which limits the generalizability of our findings to other age groups, individuals with lower educational attainment, or those with limited technological literacy. Additionally, while it is critical to tailor advocacy interventions to the specific context for them to be effective [10], the Hong Kong context may influence specific preferences that might not generalize to other cultural settings. Another limitation is the uneven gender distribution, with female participants comprising 75% of our sample, which may underrepresent the unique perspectives and needs of survivors of other genders. Furthermore, we did not specify a particular platform purpose when asking participants about their preferences. Rather than presenting a predefined scenario (e.g., a platform focused on identifying abusive behaviors, safety planning, or connecting with resources), we asked about DV-related digital platforms and AI chatbots for help-seeking initiatives. While this open-ended approach allowed us to capture a broad range of survivor perspectives without constraining their input, the resulting recommendations are not tailored to specific platform functions. Future research should investigate survivor preferences within more specific use-case scenarios to generate more targeted design guidelines.

The rapidly evolving nature of AI technology also presents a limitation, as participants’ perspectives reflect their understanding of current capabilities rather than potential future developments. Longitudinal research tracking changing perceptions as these technologies evolve would provide valuable insights for ongoing development efforts. Future research should also include participatory design studies that actively involve DV survivors in prototype development and testing. Such approaches would provide more concrete feedback on specific implementations of the features recommended in this study. Additionally, while this study focused on adult survivors’ perspectives, participants highlighted the need for age-appropriate digital resources for children and adolescents who may not recognize abusive behaviors. Future research should explore the specific needs of developing such platforms for children and adolescents, with distinct design considerations tailored to them.

## Conclusions

Participants who have experienced DV highlighted preferences for digital platforms and AI chatbots for help-seeking, particularly with respect to content, functions, and format. While AI chatbots were valued for improving accessibility, filtering information, and offering nonjudgmental feedback, concerns about emotional insufficiency, lack of personalization, and misinformation were noted. This study underscores the importance of trauma-informed design and positions AI as a complementary tool rather than a replacement for human support. Future efforts should adopt participatory approaches to ensure interventions meet the evolving needs of DV survivors across diverse contexts.

## Supporting information

S1 Appendix Semi-structured interview guide. (DOCX)

S2 Appendix Thematic analysis and coding process. (DOCX)

S1 File Ethical Approval. (PDF)

## References

[1] World Health Organization. Global status report on violence prevention 2014. 2014.

[2] Choi AW-M, Lo BC-Y, Lo RT-F, To PY-L, Wong JY-H. Intimate Partner Violence Victimization, Social Support, and Resilience: Effects on the Anxiety Levels of Young Mothers. J Interpers Violence. 2021;36(21–22):NP12299–323. doi: 10.1177/0886260519888532 31789087

[3] Ennis N, Sijercic I, Monson CM. Trauma-focused cognitive-behavioral therapies for posttraumatic stress disorder under ongoing threat: A systematic review. Clin Psychol Rev. 2021;88:102049. doi: 10.1016/j.cpr.2021.102049 34139653

[4] Kim B, Merlo AV. Domestic Homicide: A Synthesis of Systematic Review Evidence. Trauma Violence Abuse. 2023;24(2):776–93. doi: 10.1177/15248380211043812 34510978

[5] Ramesh R, Yue JK, Manley GT, Tarapore PE, DiGiorgio AM. Epidemiology of Intimate Partner and Domestic Violence-Related Traumatic Brain Injury in the United States, 2018 to 2021: A National Trauma Data Bank Cohort Analysis of 3891 Patients. Neurosurgery. 2024;95(5):1135–47. doi: 10.1227/neu.0000000000002983 38747596

[6] Wright EN, Hanlon A, Lozano A, Teitelman AM. The Association Between Intimate Partner Violence and 30-Year Cardiovascular Disease Risk Among Young Adult Women. J Interpers Violence. 2021;36(11–12):NP6643–60. doi: 10.1177/0886260518816324 30522391

[7] Hong C, Wang Y, Wang Y, Pushpanadh S, Stephenson R, Keum BT, et al. The Associations Between Intimate Partner Violence and Mental Health Outcomes Among Sexual Minority Men: A Systematic Review and Meta-Analysis. Trauma Violence Abuse. 2025;26(1):58–72. doi: 10.1177/15248380241275976 39468405

[8] Sheeran N, Jenkins A, Humphreys T, Ter Horst S, Higgins M. Investigating the Impact of Reproductive Coercion and Intimate Partner Violence on Psychological and Sexual Wellbeing. J Interpers Violence. 2025;40(3–4):726–55. doi: 10.1177/08862605241253026 38752449 PMC11673295

[9] Dufour GK, Gerhardt E, McArthur J, Ternes M. Help-Seeking Behavior and Domestic Violence. Encyclopedia of Domestic Violence. Springer International Publishing. 2023:1–13. doi: 10.1007/978-3-030-85493-5_741-1

[10] Rivas C, Vigurs C, Cameron J, Yeo L. A realist review of which advocacy interventions work for which abused women under what circumstances. Cochrane Database Syst Rev. 2019;6(6):CD013135. doi: 10.1002/14651858.CD013135.pub2 31254283 PMC6598804

[11] Saint Arnault D, Zonp Z. Understanding help-seeking barriers after Gender-Based Violence: Validation of the Barriers to Help Seeking-Trauma version (BHS-TR). Arch Psychiatr Nurs. 2022;37:1–9. doi: 10.1016/j.apnu.2021.12.004 35337432

[12] Naismith I, Ripoll-Nuñez K, Henao GB. Depression, Anxiety, and Posttraumatic Stress Disorder Following Intimate Partner Violence: The Role of Self-Criticism, Guilt, and Gender Beliefs. Violence Against Women. 2024;30(3–4):791–811. doi: 10.1177/10778012221142917 36482687

[13] Gilbert D, Postel EB. Truth without trauma: reducing re-traumatization throughout the justice system. University of Louisville Law Review. 2021;60.

[14] Nayak SS, Efimov X, Ncube CN, Griffith J, Molnar BE. “No Safe Spaces”: The Retraumatization and Dehumanization of Immigrant Survivors of Domestic Violence in the United States. Journal of Immigrant & Refugee Studies. 2023;24(1):158–73. doi: 10.1080/15562948.2023.2278055

[15] Lam TP, Chan HY, Piterman L, Wong SYS, Lam KF, Sun KS. Factors that facilitate recognition and management of domestic violence by primary care physicians in a Chinese context - a mixed methods study in Hong Kong. BMC Fam Pract. 2020;21:155. doi: 10.1186/s12875-020-01228-4 32731852 PMC7394675

[16] Hui V, Zhang B, Jeon B, Wong KCA, Klem ML, Lee YJ. Harnessing Health Information Technology in Domestic Violence in the United States: A Scoping Review. Public Health Rev. 2024;45:1606654. doi: 10.3389/phrs.2024.1606654 38974136 PMC11224144

[17] Hui V, Constantino RE, Lee YJ. Harnessing Machine Learning in Tackling Domestic Violence-An Integrative Review. Int J Environ Res Public Health. 2023;20(6):4984. doi: 10.3390/ijerph20064984 36981893 PMC10049304

[18] Storer HL, Nyerges EX, Hamby S. Technology “Feels Less Threatening”: The processes by which digital technologies facilitate youths’ access to services at intimate partner violence organizations. Children and Youth Services Review. 2022;139:106573. doi: 10.1016/j.childyouth.2022.106573

[19] Emezue C, Chase J-AD, Udmuangpia T, Bloom TL. Technology-based and digital interventions for intimate partner violence: A systematic review and meta-analysis. Campbell Syst Rev. 2022;18(3):e1271. doi: 10.1002/cl2.1271 36909881 PMC9419475

[20] Glass NE, Clough A, Messing JT, Bloom T, Brown ML, Eden KB, et al. Longitudinal Impact of the myPlan App on Health and Safety Among College Women Experiencing Partner Violence. J Interpers Violence. 2022;37(13–14):NP11436–59. doi: 10.1177/0886260521991880 33576291

[21] Decker MR, Wood SN, Hameeduddin Z, Kennedy SR, Perrin N, Tallam C, et al. Safety decision-making and planning mobile app for intimate partner violence prevention and response: randomised controlled trial in Kenya. BMJ Glob Health. 2020;5(7):e002091. doi: 10.1136/bmjgh-2019-002091 32675229 PMC7368487

[22] Hegarty K, Tarzia L, Valpied J, Murray E, Humphreys C, Taft A, et al. An online healthy relationship tool and safety decision aid for women experiencing intimate partner violence (I-DECIDE): a randomised controlled trial. Lancet Public Health. 2019;4(6):e301–10. doi: 10.1016/S2468-2667(19)30079-9 31155223

[23] Koziol-McLain J, Vandal AC, Wilson D, Nada-Raja S, Dobbs T, McLean C. Efficacy of a web-based safety decision aid for women experiencing intimate partner violence: randomized controlled trial. J Med Internet Res. 2018;20(1):e8. 10.2196/jmir.8617 PMC6858022 29321125

[24] Ford-Gilboe M, Varcoe C, Scott-Storey K, Perrin N, Wuest J, Wathen CN, et al. Longitudinal impacts of an online safety and health intervention for women experiencing intimate partner violence: randomized controlled trial. BMC Public Health. 2020;20(1):260. doi: 10.1186/s12889-020-8152-8 32098633 PMC7043036

[25] Shaygan M, Hosseini FA, Shemiran M, Hedayati A. The effect of mobile-based logotherapy on depression, suicidal ideation, and hopelessness in patients with major depressive disorder: a mixed-methods study. Sci Rep. 2023;13(1):15828. doi: 10.1038/s41598-023-43051-8 37740006 PMC10516998

[26] Bartal A, Jagodnik KM, Chan SJ, Dekel S. OpenAI’s Narrative Embeddings Can Be Used for Detecting Post-Traumatic Stress Following Childbirth Via Birth Stories. Res Sq. 2024. doi: 10.21203/rs.3.rs-3428787/v2 38605073 PMC11009279

[27] Held P, Stade EC, Dondanville K, Wiltsey Stirman S. Generative artificial intelligence in posttraumatic stress disorder treatment: Exploring five different use cases. J Trauma Stress. 2025;38(5):813–20. doi: 10.1002/jts.23188 40736259 PMC12551617

[28] Feng X, Tian L, Ho GWK, Yorke J, Hui V. The Effectiveness of AI Chatbots in Alleviating Mental Distress and Promoting Health Behaviors Among Adolescents and Young Adults: Systematic Review and Meta-Analysis. J Med Internet Res. 2025;27:e79850. doi: 10.2196/79850 41313175 PMC12661615

[29] Guo Z, Lai A, Thygesen JH, Farrington J, Keen T, Li K. Large Language Models for Mental Health Applications: Systematic Review. JMIR Ment Health. 2024;11:e57400. doi: 10.2196/57400 39423368 PMC11530718

[30] Yuan A, Garcia Colato E, Pescosolido B, Song H, Samtani S. Improving Workplace Well-being in Modern Organizations: A Review of Large Language Model-based Mental Health Chatbots. ACM Trans Manage Inf Syst. 2025;16(1):1–26. doi: 10.1145/3701041

[31] Guan S, Hui V, Stiglic G, Constantino RE, Lee YJ, Wong AKC. Classifying the Information Needs of Survivors of Domestic Violence in Online Health Communities Using Large Language Models: Prediction Model Development and Evaluation Study. J Med Internet Res. 2025;27:e65397. doi: 10.2196/65397 40354642 PMC12107195

[32] Silva T, Agampodi T, Evans M, Knipe D, Rathnayake A, Rajapakse T. Barriers to help-seeking from healthcare professionals amongst women who experience domestic violence - a qualitative study in Sri Lanka. BMC Public Health. 2022;22(1):785. doi: 10.1186/s12889-022-13116-w 35410170 PMC9004164

[33] Mattson S, Shearer N, Long C. Exploring telehealth opportunities in domestic violence shelters. J Am Acad Nurse Pract. 2002;14(10):465–70. doi: 10.1111/j.1745-7599.2002.tb00077.x 12426804

[34] Liang B, Goodman L, Tummala-Narra P, Weintraub S. A theoretical framework for understanding help-seeking processes among survivors of intimate partner violence. Am J Community Psychol. 2005;36(1–2):71–84. doi: 10.1007/s10464-005-6233-6 16134045

[35] Hui V, Eby M, Constantino RE, Lee H, Zelazny J, Chang JC, et al. Examining the Supports and Advice That Women With Intimate Partner Violence Experience Received in Online Health Communities: Text Mining Approach. J Med Internet Res. 2023;25:e48607. doi: 10.2196/48607 37812467 PMC10594147

[36] Braun V, Clarke V. Using thematic analysis in psychology. Qualitative Research in Psychology. 2006;3(2):77–101. doi: 10.1191/1478088706qp063oa

[37] Braun V, Clarke V. Reflecting on reflexive thematic analysis. Qualitative Research in Sport, Exercise and Health. 2019;11(4):589–97. doi: 10.1080/2159676x.2019.1628806

[38] Braun V, Clarke V. One size fits all? What counts as quality practice in (reflexive) thematic analysis? Qual Res Psychol. 2021;18(3):328–52.

[39] Chandler RD, Warner S, Guillaume D, Wells J. Exploring ChatGPT’s role in women’s health self-education: A descriptive study comparing responses with public health guidance. Nurs Outlook. 2025;73(4):102468. doi: 10.1016/j.outlook.2025.102468 40614295 PMC12233143

[40] Siddals S, Coxon A, Torous J. “It just happened to be the perfect thing”: Real-life experiences of generative AI chatbots for mental health. Springer Science and Business Media LLC. 2024. doi: 10.21203/rs.3.rs-4612612/v1 PMC11514308 39465310

[41] Saqib H, Sumyia, Saqib A. AI Chatbots And Psychotherapy: A Match Made In Heaven?. J Pak Med Assoc. 2023;73(11):2321. doi: 10.47391/JPMA.9608 38013574

[42] Pham KT, Nabizadeh A, Selek S. Artificial intelligence and chatbots in psychiatry. Psychiatric Quarterly. 2022;93:249–53. doi: 10.1007/S11126-022-09973-8 35212940 PMC8873348

[43] Nadarzynski T, Miles O, Cowie A, Ridge D. Acceptability of artificial intelligence (AI)-led chatbot services in healthcare: A mixed-methods study. Digit Health. 2019;5:2055207619871808. doi: 10.1177/2055207619871808 31467682 PMC6704417

[44] Altamimi I, Altamimi A, Alhumimidi AS, Altamimi A, Temsah M-H. Artificial Intelligence (AI) Chatbots in Medicine: A Supplement, Not a Substitute. Cureus. 2023;15(6):e40922. doi: 10.7759/cureus.40922 37496532 PMC10367431

[45] Cheng Y, Jiang H. AI‐Powered mental health chatbots: Examining users’ motivations, active communicative action and engagement after mass‐shooting disasters. Contingencies & Crisis Mgmt. 2020;28(3):339–54. doi: 10.1111/1468-5973.12319

[46] Miner AS, Shah N, Bullock KD, Arnow BA, Bailenson J, Hancock J. Key Considerations for Incorporating Conversational AI in Psychotherapy. Front Psychiatry. 2019;10:746. doi: 10.3389/fpsyt.2019.00746 31681047 PMC6813224

[47] Rubin M, Arnon H, Huppert JD, Perry A. Considering the Role of Human Empathy in AI-Driven Therapy. JMIR Ment Health. 2024;11:e56529. doi: 10.2196/56529 38861302 PMC11200042

[48] Awuah WA, Aderinto N, Poornaselvan J, Tan JK, Shah MH, Ashinze P, et al. Empowering health care consumers & understanding patients’ perspectives on AI integration in oncology and surgery: A perspective. Health Sci Rep. 2024;7(7):e2268. doi: 10.1002/hsr2.2268 39050906 PMC11266117

[49] Benda N, Desai P, Reza Z, Zheng A, Kumar S, Harkins S, et al. Patient Perspectives on AI for Mental Health Care: Cross-Sectional Survey Study. JMIR Ment Health. 2024;11:e58462. doi: 10.2196/58462 39293056 PMC11447436

[50] Hogg HDJ, Al-Zubaidy M, Technology Enhanced Macular Services Study Reference Group, Talks J, Denniston AK, Kelly CJ, et al. Stakeholder Perspectives of Clinical Artificial Intelligence Implementation: Systematic Review of Qualitative Evidence. J Med Internet Res. 2023;25:e39742. doi: 10.2196/39742 36626192 PMC9875023

[51] Abdulai A-F, Naghdali H, Tekie Ghirmay E, Adam F, Bawafaa E. Trauma-Informed Care in Digital Health Technologies: Protocol for a Scoping Review. JMIR Res Protoc. 2023;12:e46842. doi: 10.2196/46842 37351935 PMC10337410

[52] Williams K, Harb M, Satyen L, Davies M. s-CAPE trauma recovery program: the need for a holistic, trauma- and violence-informed domestic violence framework. Front Glob Womens Health. 2024;5:1404599. doi: 10.3389/fgwh.2024.1404599 39574835 PMC11578950

[53] Owen C, Crane J. Trauma-Informed Design of Supported Housing: A Scoping Review through the Lens of Neuroscience. Int J Environ Res Public Health. 2022;19(21):14279. doi: 10.3390/ijerph192114279 36361166 PMC9658651

