# Supplementary material for: Perspectives and preferences of domestic violence survivors regarding digital platform and AI chatbot for help-seeking: A qualitative study
Source: PLoS One. 2026 Feb 23;21(2):e0342453. doi: 10.1371/journal.pone.0342453 (PMC12928437; doi:10.1371/journal.pone.0342453)
Supplement: S1 Appendix — (DOCX) [file pone.0342453.s001.docx]

**INTERVIEW GUIDE**

**Introduction**

1. Introduction and provision of project information sheet
2. Consent form (signature required) and audio recording permission
3. Reminder: You may stop the interview at any time if you feel uncomfortable

**Part I: DV experience**

**1. Can you share about your experience with domestic violence in a way that feels comfortable for you?**

*Prompts:*

1. What types of abuse have you experienced? (physical harm, emotional distress, financial control, other concerns)
2. when was the last time with DV experience? and who is the perpetrators/victims?
3. How have these experiences affected your mental health/physical health?
4. How did you cope with it usually?

**2. Have you sought support or help for these experiences?**

*Prompts:*

1. If yes: What types of support did you try? (counseling, hotline, legal services, shelter, online resources)
2. What was helpful? What challenges did you encounter?
3. If no: What prevented you from seeking help at that time?

**Part II: Digital Platform Features**

[Interviewer shows example platform (1800RESPECT) and allows 5 minutes for exploration]

**1. What would you expect from an online digital platform designed for domestic violence survivors?**

*Prompts:*

1. How important is anonymity to you when using such a platform?
2. What concerns about privacy or safety would you have?
3. Would you prefer interactive features (e.g., forums, live chat)? Why or why not?
4. What kind of supportive features would be most helpful for you?

**2. Do you think a private and safe digital platform could facilitate your help-seeking? Please explain.**

*Prompts:*

1. What does "private" mean to you in this context?
2. If any, would you have about using such a platform?

**3. How would you prefer information to be provided on a digital platform?**

*Prompts:*

1. In what ways should the information be presented (e.g., text, video, infographics)?
2. What format would be easiest for you to understand and use?
3. How much detail would you want in the information provided?

**4. What type of content would you browse the most on a digital platform?**

*Prompts:*

1. Would you be more interested in educational resources, peer support, professional advice, or other content?
2. What topics would be most relevant to your needs?

**5. Can you share any previous experiences with online platforms or resources for help-seeking?**

*Prompts:*

1. What worked well? What did not work well?
2. Based on your experience, what expectations do you have for future platforms?

**Part III: AI Chatbot Features**

[Interviewer provides brief explanation of AI chatbots and shows example (e.g., GPT 3.5 / Llama 3)]

**1. What are your thoughts on using AI-assisted features (e.g., chatbots) for help-seeking related to domestic violence?**

*Prompts:*

1. Have you ever used an AI chatbot before? If so, what was your experience?
2. What benefits do you see in using AI chatbots for DV-related support?
3. What concerns or limitations do you perceive?

**2. Would you be comfortable using an AI chatbot for screening purposes (e.g., identifying red flags in relationships)?**

*Prompts:*

1. What information would you feel comfortable sharing with an AI chatbot?
2. What concerns would you have about accuracy or privacy?

**3. How do you think AI chatbots should work together with human professionals?**

*Prompts:*

1. In what situations should an AI chatbot refer you to a human counselor?
2. What would make you trust an AI chatbot to provide safe and accurate information?

**4. What specific features or safeguards would you want to see in an AI chatbot designed for DV support?**

*Prompts:*

1. How should it handle crisis situations?
2. What reassurances about confidentiality would you need?
3. Would official endorsement (e.g., by government or DV organizations) increase your trust?

**Closing Question**

1. **Is there anything else about digital platforms or AI chatbots for DV support that we haven't discussed but you think is important to share?**

***Note:*** *This interview guide is semi-structured. Interviewers may use follow-up questions and probes based on participant responses to explore topics in greater depth. The order of questions may be adjusted based on the natural flow of the conversation.*
