# Supplementary material for: Perspectives and preferences of domestic violence survivors regarding digital platform and AI chatbot for help-seeking: A qualitative study
Source: PLoS One. 2026 Feb 23;21(2):e0342453. doi: 10.1371/journal.pone.0342453 (PMC12928437; doi:10.1371/journal.pone.0342453)
Supplement: S2 Appendix — (DOCX) [file pone.0342453.s002.docx]

**Supplementary Table 1: Thematic Analysis of Recommended Features for Digital Platforms with Representative Participant Quotes**

| **Theme 1 Recommended features for digital platform** | | **Transcripts Descriptions** |
| --- | --- | --- |
| 1. **Content of platform** | |  |
|  | Evidence-based information | - I like looking at things with data or maybe content from literature, which I find helpful [p03] - I enjoy reading things with numbers and data, like research reports [p04] - Data is helpful! I really want data [p05] - I'm interested in statistics about child-rearing, or spousal abuse, or sexual violence [p26] |
|  | Step-by-step practical guidance | - I hope this information can provide more substantial assistance, like knowing the immediate steps I need to take [p35]. - I want more practical steps, like what I need to do to file for divorce, step by step, or how to teach children to accept this family situation, or how to accept their past [p10] - It's really important to have a step-by-step process, though such steps might not apply to every person's case, but it still serves as a useful reference [p32] |
|  | Shared experiences from previous DV survivors | - When I search for these keywords, it might bring up cases where a group of people have faced similar situations. Maybe it would show "Mr. A's story" first, and how he solved his problem [p01] - I'd like to know about people who experienced domestic violence as children, and what their mental state might be like when they reach their thirties or forties, or how they face building their own families [p03] - Something that could be highly interactive, where I can see the experiences of other survivors or victims, especially seeing the results after they sought help. That might increase my motivation [p05] |
|  | DV Knowledge and awareness | - I think it could be like those simulation clips showing how domestic violence happens in a household [p01]. - I think it should provide more information on how to define domestic violence and make it clearer [p26]. - The definition of domestic violence should be more detailed, categorized into physical violence, verbal violence, sexual violence, cold violence, and not just simply grouped under domestic violence [p34] |
|  | Self-assessment on DV | - If really designing a website, I think there should be some tests for them to do. I think these screening forms are very important, especially for children, to track if they have experienced domestic violence through their behavior, to let them know what they're experiencing [p27] |
|  | Relevant local DV context | - The information and titles should clearly indicate that they are specifically for Hong Kong [p18] - I'm more curious about Hong Kong-specific information [p28] - I noticed that a lot of the information is from Taiwan, which isn't very relevant to Hong Kong [p17] |
| 1. **Functions of platform** | |  |
|  | Anonymity and Privacy | - If the other person doesn't mind, they can go public if they want to, or use a pseudonym if they prefer [p02] - What we should avoid is requiring full Chinese names, date of birth, or anything that can identify you [p04] - Posts can be written anonymously [p25] - Having a password makes it very confidential, so others can't log in to see it [p32] |
|  | Personal Incident documentation and reporting autonomy | - Maybe provide a simple Google Form where you can fill in what harm you experienced, how many times it happened, who the perpetrator was, when it happened, and then leave your contact information, so someone can contact you or your parents [p10] - There needs to be a place where help-seekers can write down what happened. Then they can choose whether they need immediate assistance [p30] |
|  | Emergency Support and response | - I think they did well with allowing immediate contact and stating it's 24-hour service. I think this method is better because you can get an immediate response or clear guidance [p16] - Help-seeking needs to be quick. Whether it's immediate online support or direct access to hotline numbers. It needs to be immediate because people are usually urgent when looking for these things [p18] - If their website had something like a Q&A section similar to other websites, I think that would be helpful [p20] |
|  | Peer Support Network | - They could hire or find volunteers who have experienced these things themselves to be comforters [p23] - Let victims or survivors share their stories. Then everyone can support each other, but this function needs to be promoted so that people who need it can find this channel [p08] - This platform should have a place for them to write about their own experiences, how they got through it. Our community members can share their experiences together, share how they feel about living in the center. I think that's really good, through these channels [p10] |
|  | Official Endorsement and Healthcare Professional support | - Having government agencies endorse this, whether it's the police department or even the Social Welfare Department [p16] - You have an anonymous platform to talk about your problems, with social workers who can help. At least they know how to handle the situation, because that's important [p14] - Maybe this platform could have a registered social worker or registered nurse who can respond immediately [p06] |
|  | Supportive and safe online health community | - There should be a place for other survivors or victims to write about their experiences and how they recovered [p10] - It's like having a discussion forum, but most importantly, it needs moderators. This prevents people from attacking each other or triggering each other's trauma. When talking about these topics, people's emotions tend to be more sensitive, and inner wounds can easily be triggered. Therefore, someone needs to manage the order [p16] - The platform should probably have someone to talk with them, or to link them up with resources [p05] - They could hire or find volunteers who have experienced these things themselves to be comforters [p23] |
| 1. **Format of platform** | |  |
|  | Procedural visualization | - Should have diagrams showing the steps - like step 1 is making a call, step 2 is police report, and so on. You can click each step for more details [p09] |
|  | Engaging multimedia contents | - Like those simulation clips showing how domestic violence might happen [p01] - It would be better if there were animations, videos, and background music [p02] - Find some actors to simulate the mindset of domestic violence victims and perpetrators, and then their housing and financial issues can be brief but hit the key points [p11] - It shouldn't be just text, but rather a combination of images and text telling you what you should do [p16]. - Maybe use pictures, and try to break down the information into smaller pieces [p28]. |
|  | Calming and approachable Visual Design | - I'd feel more comfortable reading text that contains more emotional support [p03] - The screen should look more relaxing, maybe with green colors [p02] - More colorful, and with some reliable logos [p03] - Colors matter too. Maybe more neutral, lighter colors that don't give people an aggressive feeling [p05] - But not too colorful, since this isn't a happy topic, and no black as it feels too tragic [p14] |
|  | Text Layout | - I don't want it to be just a wall of text. At minimum, it should separate topics by type and address key points in detail [p11] - "Emergency Hotline" should be in very large font, in a very obvious position [p14] - Although it's in list form like bullet points, if people see a long list like 1,2,3,4,5 but each point is still a large paragraph, it's not concise enough [p18] |

**Supplementary Table 2: Thematic Analysis of Perspectives on AI Chatbot Integration with Representative Participant Quotes**

| **Theme 2 Perspectives on AI** | | **Transcripts Descriptions** |
| --- | --- | --- |
| 1. **Perceived Benefits** | |  |
|  | Information filtering and summarization | - I find it quite challenging to filter through so much information online, so it would be good if there was a method to help filter the information [p16]. - I think using something like ChatGPT or similar AI robots to help streamline information for them would work well [p37]. - The chatbot can summarize and help you find the information you want, instead of googling and slowly typing keywords to search [p34]. - If ChatGPT could summarize everything for me, it would at least quickly list all the more important points, which would make it convenient for me to know immediately without having to search for a long time. I could also ask follow-up questions without needing to read through detailed texts about how to seek help [p33]. |
|  | Non-judgmental feedback | - ChatGPT's best aspect is being non-judgmental, as it can really speak to you in a very neutral way [p28] - It won't judge me. Its evaluation is truly as a third party, it won't have any bias, because when it speaks it seems a bit careful not to offend you, or it gives rational analysis from different angles [p34] |
|  | Accessibility to broad users | - Some victims might not have the patience to read online information, or elderly people might not be literate. Through AI, demonstrations and explanations might be clearer and more interesting [p11] - ChatGPT is relatively simple in that the knowledge requirement isn't that high. You might ask a question, and as long as you can type, that's OK [p13] |
| 1. **Perceived Risks** | |  |
|  | Insufficient emotional support | - Because ultimately a robot just has numbers programmed into it, robots don't have emotions and haven't experienced anything firsthand [p01] - An AI just summarizes information from the internet and throws a few words at me, so it lacks that warmth [p03] - It's still just a robot, not as warm as talking to a real person [p04] - I don't think artificial intelligence can necessarily fulfill an emotional support role [p05] - AI really can't replace psychologists and social workers. I think it can help you find a website map more quickly, but responding to emotions would be difficult [p27] - When you're very upset and talking about your emotions, at the beginning it will say it's just an AI and doesn't understand you. But I think the responses it gives, on the flip side, don't have emotions [p34] |
|  | Misleading information | - I worry about the credibility of information provided by AI. Because its current search engine might sometimes combine information from different countries, it might give a helpline number that can't be dialed in Hong Kong. I really worry about the accuracy of its information [p05] - I used ChatGPT to find information for me, but the results it gave me were fake [p13]. - I think if you're talking with them, with these chatbots, the information might be somewhat inaccurate [p37] |
|  | Lack of personalization | - When I first started using AI, I felt it wasn't very personalized [p28] - If the case is very simple, then I believe ChatGPT can teach you what to do. Most times human-to-human issues might be the most complex and difficult to handle. Maybe when you ask ChatGPT, everyone might get almost the same information, so such a one-size-fits-all approach might not really work [p32] |
|  | Outdated information | - It's still using a lot of outdated data [p15] - Because I've used ChatGPT myself, but it's not real-time - its database gets updated, so the information you can find is actually limited. That's why I haven't really used it for searching [p34] |
| 1. **Recommended function on AI chatbot** | |  |
|  | Crisis detection through keywords and notifications | - Suppose you might type certain words, then it's a high red flag, meaning I know this case is very likely to be, for example, suicide [p15] - Perhaps when the system sees victims writing messages that show a lot of low self-esteem, or even suicidal tendencies. This system could use AI to identify these high-risk individuals [p02] - I think if there were some identifying keywords, for example if a victim mentions serious adverse events, life-threatening situations, or if the AI system detects phrases like "I really want to die" or "I really want to commit suicide," these keywords might trigger a notification asking "Would you like to communicate with a real person for help?" [p37] |
|  | AI as non-judgmental counseling support and disclosure | - I even think that if AI counseling develops better, it might know what to say at the right moment, which could make it even more sensitive to people's needs [p18] - I've heard that some people around me, or friends of friends, would set keywords for these AI chatbots to treat them like therapists or something similar [p37] - I understand why some people would want to use ChatGPT for counseling, because they might not want to discuss their issues with a real person. The best aspect of ChatGPT in this regard is that it's non-judgmental - it can really speak to you in a very neutral way [p28] |
|  | Referral service and Integration with professional support | - AI could function more like a personal emergency alarm system. There could be a platform with AI capabilities that can detect when there might actually be a fatal incident about to occur, then social workers would be alerted through the AI and could immediately make a phone call [p01] - It would be good if someone talks to AI first and then gets referred to relevant professionals. This helps them understand how to properly seek help and express what they want to do [p23] - Having real people as backup is an important function [p37] |

**Supplementary Table 3: Illustrative Example of the Coding Process from Raw Quote to Theme**

| **Raw Quote** | **Code** | **Subtheme** | **Theme** |
| --- | --- | --- | --- |
| "I enjoy reading things with numbers and data, like research reports" [p04] | Preference for data-backed content | Evidence-based information | Content of platform |
| "I want more practical steps, like what I need to do to file for divorce, step by step" [p10] | Need for actionable guidance | Step-by-step practical guidance | Content of platform |
| "I think it should provide more information on how to define domestic violence and make it clearer" [p26] | Desire for clearer DV definitions | DV knowledge and awareness | Content of platform |
