# Supplementary material for: Perspectives and preferences of domestic violence survivors regarding digital platform and AI chatbot for help-seeking: A qualitative study
Source: PLoS One. 2026 Feb 23;21(2):e0342453. doi: 10.1371/journal.pone.0342453 (PMC12928437; doi:10.1371/journal.pone.0342453)
Supplement: S1 File — (PDF) [file pone.0342453.s003.pdf]

THE HONG KONG POLYTECHNIC UNIVERSITY
香港理工大學

To	Hui Chi Ching Vivian (School of Nursing)
From	Leung Yee Man Angela, Chair, Departmental Research Committee
Email	angela.ym.leung@polyu.edu.hk Date 24-Mar-2023

Application for Ethical Review for Teaching/Research Involving Human Subjects

I write to inform you that approval has been given to your application for human subjects ethics review of the following project for a period from 06-Feb-2023 to 05-Feb-2026:

**Project Title:**	Exploring the online help-seeking and health information-seeking behavior among people with domestic violence experiences in Hong Kong
**Department:**	School of Nursing
**Principal Investigator:**	Hui Chi Ching Vivian
**Project Start Date:**	06-Feb-2023
**Project type:**	Human subjects (non-clinical)
**Reference Number:**	HSEARS20230111007

You will be held responsible for the ethical approval granted for the project and the ethical conduct of the personnel involved in the project. In case the Co-PI, if any, has also obtained ethical approval for the project, the Co-PI will also assume the responsibility in respect of the ethical approval (in relation to the areas of expertise of respective Co-PI in accordance with the stipulations given by the approving authority).

You are responsible for informing the PolyU Institutional Review Board in advance of any changes in the proposal or procedures which may affect the validity of this ethical approval.

Leung Yee Man Angela

Chair

Departmental Research Committee (on behalf of PolyU Institutional Review Board)
